# Obstructive teratoma in the right ventricle of a newborn: a case report

**DOI:** 10.1186/s13019-019-0874-2

**Published:** 2019-03-12

**Authors:** Yasser Farid, Louis Chebli, Valérie Seghers, Wendy Dewals, Ahmed Sanoussi, Pierre Wauthy

**Affiliations:** 1 Department of Cardiac Surgery, Hôpital Universitaire des Enfants Reine Fabiola, Université Libre de Bruxelles (ULB), 15 Avenue J Crocq, B-1020 Laeken, Brussels, Belgium; 2 Department of Pathology, Hôpital Universitaire des Enfants Reine Fabiola, Université Libre de Bruxelles (ULB), 15 Avenue J Crocq, B-1020 Laeken, Brussels, Belgium; 3 Department of Cardiology, UZ Hospital Brussels, Jette Campus Avenue du Laerbeek 101, 1090 Jette, Belgium

**Keywords:** Congenital heart disease, Congenital heart surgery, Cardiac tumors, Neonate, Surgery/incisions

## Abstract

**Background:**

A newborn was diagnosed by echocardiogram with an asymptomatic cardiac mass in the right ventricle after a systolic cardiac murmur was detected at birth.

**Case presentation:**

Nine days after birth, the newborn presented with three syncopal episodes and oxygen desaturation which required resuscitation. The mass induced a complete right ventricular outflow tract obstruction. The presence of a patent foramen oval and a patent ductus arteriosus explained the absence of symptoms at birth. Surgery was rapidly considered since the situation was life threatening. The tumor was successfully resected. The mass was a mature teratoma confirmed by microscopic examination, illustrated by pictures and video.

**Conclusions:**

This case was unique because of the absence of symptoms in the first 9 days of the newborn’s life even though symptoms should have mounted due to the obstruction postpartum. The delay was correlated to the closure of the patent ductus arteriosus. It is recommended that newborns with any cardiac mass be followed up regularly due to hemodynamic changes at birth**.**

**Electronic supplementary material:**

The online version of this article (10.1186/s13019-019-0874-2) contains supplementary material, which is available to authorized users.

## Background

Primary cardiac tumors in newborns are extremely rare and more than 90% of them are benign. They occur approximately in 1 every 100,000 live births [1]. The most frequent tumors of the heart observed in pediatric patients are rhabdomyomas, fibromas and lipomas. Rhabdomyoma and teratoma represent more than 70% of the primary tumors of the heart in fetuses and neonates [1, 2]. The main clinical presentation in the foetus or neonate relate to the mass effect of the tumor and to the accumulation of fluid in the pericardial space. Congestive heart failure, respiratory distress and cyanosis are predominant signs in the neonate [2] .We report a clinical history of a newborn with right ventricular outflow tract obstruction with unusual asymptomatic beginnings.

## Case presentation

A 24 years old female delivered a female baby at 38 weeks through cesarean. The baby’s appearance, pulse, grimace, activity, and respiration (APGAR) score at delivery, and at 5 min was normal. Postpartum, a pulmonary systolic murmur was detected during a routine clinical exam. An echocardiogram was performed and showed the presence of a tumor in the right ventricle. The baby was kept under supervision. Her hemodynamic parameters remained stable and presented no symptoms, hence, she was discharged. Nine days later, while the baby was being breastfed, she had a presyncopal episode, shortness of breath and grunting. All of which suddenly resolved after a few minutes. She was brought to the emergency room where she had a second episode.

During examination, her oxygen saturation was 24% in room air. She was resuscitated with mask and balloon until she stabilized and was subsequently hospitalized. The intensivist decided to do a cardiac and cerebral magnetic resonance imaging (MRI) when a third presyncopal episode occurred on the table. She was resuscitated for a second time with success. An echocardiogram was performed and for the first time, it showed cyclic complete obstruction of the right ventricular outflow tract. The recorded echocardiogram is shown in Additional file 1: Video S1.

Furthermore, the MRI showed a mobile and large mass in the right ventricle which measured 13 mm × 9 mm. The mass was attached to the baso septum of the right ventricle and moved completely to the pulmonary trunk during systole. Pulmonary artery flow measurements showed the presence of pulmonary insufficiency. We observed a patent foramen oval (PFO) and a patent ductus arteriosus (PDA), both of which had right to left shunts. In terms of the global kinetics of the left ventricle, it was homogeneous compared to a slight dilation in the right ventricle. Because of the previous clinical description, and more predominantly the repeated syncopal episodes that only appeared after 9 days postpartum, urgent surgery was indicated.

The surgical procedure was performed under standard cardiopulmonary bypass at systemic hypothermia. A right atriotomy was performed parallel to the atrioventricular groove which exposed the tricuspid valve. Through the valve, we exposed a right ventricular large mass attached to the septum. The mass was removed by excision of the foot like structure in the right ventricular outflow tract septum. The PFO and the PDA were surgically closed.

**Fig. 1:**
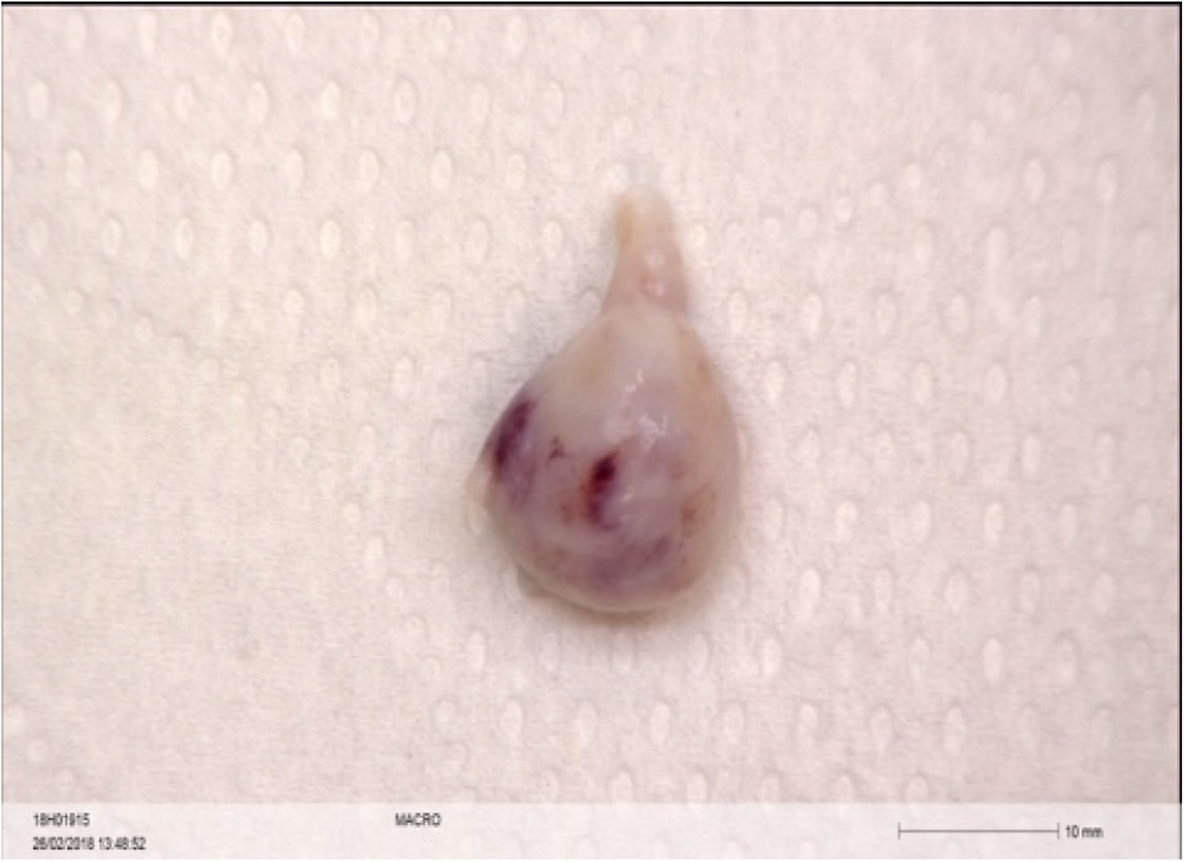
Resected teratoma with a nodular fragment of whitish coloration

**Fig. 2:**
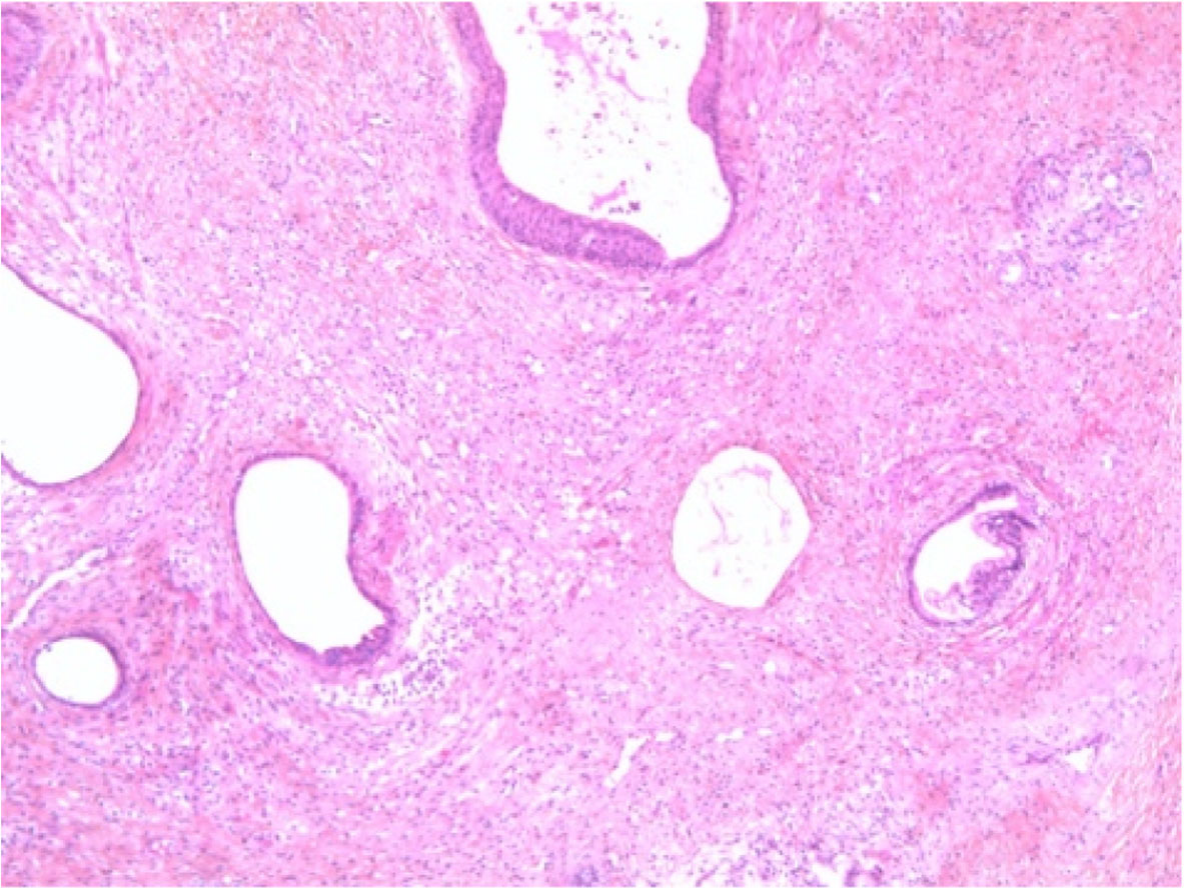
Histopathology image of a pseudo polypoid structure composed of fibrous stroma axis which contains numerous glandular structures consistent of mature teratoma

The macroscopic exam revealed that the tumor resected was a nodular mass of white discoloration which measured 1.5 cm of larger diameter for a total height of 1.7 cm. The body showed two small protuberances measuring 0 .1cm larger diameter (Fig. 1). Histopathology revealed fragments of a pseudo nodular structure composed of fibrous stroma axis which contained numerous glandular structures. An epithelial coating of variable histology was observed. Some mucosecretant glands suggested a digestive epithelium. Others had a cylindrical epithelium mucocilia, others a transitional or even squamous epithelium. There were no immature structures. The findings were consistent with a mature teratoma. (Fig. 2).

## Discussion

Cardiac tumors are extremely rare in pediatric patients and are benign in most cases [3, 4]. Rhabdomyomas are the most common ones followed by fibroma, teratoma and lipoma [5]. Severity varies hugely, some are asymptomatic, others symptomatic and few can be life threatening. Their clinical manifestation depends mostly on the anatomic location of the tumor rather than the histopathology. Different mechanisms can cause symptoms like embolization, interference with heart valves, direct invasion of the myocardium and invasion of the adjacent lung [3]. Furthermore, blood flow obstruction and rhythm disorder are frequent.

In this case report, the patient presented with a mature teratoma. Teratomas are benign tumors of embryonic origin and are the second most common ones in utero and at birth in term of frequency [1]. They are characterized by a rapid growth which can lead to serious mechanical consequences [6]. They are usually attached to the base of the great vessels in the anterior mediastinum. Other tumors like rhabdomyomas are often localized in the septum wall of the left ventricle, fibromas within the ventricular myocardium and lipomas originate in the endocardium or epicardium [6].

The teratoma was localized in the right ventricle. The size of the tumor caused a right ventricular outflow tract obstruction (Additional file 1: Video S1), with several syncopal episodes which is a typical symptom among others like peripheral edema, ascites, hepatomegaly shortness of breath and sudden death. The tumor was clearly obstructive but surprisingly symptoms only appeared after the first week of birth and not directly at birth. It is suspected that the reason of absence of symptoms is due to the presence of a PFO and PDA. Indeed, the PDA provided pulmonary perfusion in the obstructive phase of the tumor and the right ventricle was discharged by a right-left shunt through the PFO. It was a true oval foramen that could shunt from right to left by valve effect. Symptoms, and particularly, desaturation started to appear when the PDA started to close.

Whether a patient will need surgery or conservative follow up is always a topic of discussion among medical teams [7]. Surgery is usually considered for symptomatic patients which wasn’t the case in our patient at the beginning. It became so several days later when hemodynamic instability and repeated symptoms appeared.

## Conclusion

In pediatric congenital cardiac tumors, surgery is indicated depending on clear clinical manifestations and its level of severity [7]. In this unique case report, there was absence of symptoms in the first nine days of the newborn’s life. The delay was correlated to the closure of the PDA. We recommend that newborns with any cardiac mass be followed up regularly due to possible hemodynamic changes after birth, surgery should be considered within a multidisciplinary team when these changes occur.

## Additional file

Additional file 1: **Video S1.** Echocardiogram showing a cyclic complete obstruction of the right ventricular outflow tract by a large mass. (MP4 3068 kb)

## Abbreviation

APGAR: Appearance, Pulse, Grimace, Activity, and Respiration
MRI: magnetic resonance imaging
PDA: patent ductus arteriosus
PFO: patent foramen oval
